# Supplementary material for: Lack of functional STING modulates immunity but does not protect dopaminergic neurons in the alpha-synuclein pre-formed fibrils Parkinson’s disease mouse model
Source: NPJ Parkinsons Dis. 2025 Dec 11;12:17. doi: 10.1038/s41531-025-01228-0 (PMC12800057; doi:10.1038/s41531-025-01228-0)
Supplement: Supplementary file 1 — KlaestrupSupplementary_revision_2025 [file 41531_2025_1228_MOESM1_ESM.pdf]

Lack of functional STING modulates immunity but does not protect dopaminergic neurons in the alpha-synuclein pre-formed fibrils Parkinson's Disease mouse model

Ida H. Klæstrup[1,2], Line S. Reinert[1], Sara A. Ferreira[1,2], Johanne Lauritsen[1,2], Gitte U. Toft [1,2], Hjalte Gram[1,2], Poul H. Jensen[1,2], Søren R. Paludan[1] and Marina Romero-Ramos[# 1,2]

Supplementary Tables and figures

Supplementary table 1: Antibodies used for immunohistochemistry

Antibody	Dilution	Type/Clone	Company
**TH**	1:750	Polyclonal	Millipore (AB152)
**MHCII**	1:400	Monoclonal/M5/114.15.2	eBioscience (14-5321)
**MJF14**	1:25000	Monoclonal/MJFR14-6-4-2	Abcam(ab209538)
**pSer129**	1:3000	Monoclonal/D1R1R	Cell signaling technology (23706)
**P62/SQSTM1**	1:2000	Polyclonal	Nordic Biosite (18420-1-AP)
**Iba-1**	1:1000	Polyclonal	Wako Fujifilm (019-19741)
**CD68**	1:1000	Monoclonal/FA-11	Bio-Rad (MCA1957)

Supplementary table 2: Primers for biomarker assay (TaqMan gene expression assayed, Applied Biosystems).

mRNA target	Ref:
**CXCL10**	Mm00445235_m1
**CXCL1**	Mm04207460_m1
**CXCL2**	Mm00436450_m1
**CCL2**	Mm00441242_m1
**TNFα**	Mm00443260_g1
**IFN-β**	Mm00439552_s1
**MX1**	Mm00487796_m1
**IFN-γ**	Mm01168134_m1
**IL1-b**	Mm00434228_m1
**IL6**	Mm00446190_m1
**IL-10**	Mm01288386_m1
**gCSF**	Mm00438334_m1
**TREM2**	Mm04209424_g1
**C1qa**	Mm00432142_m1
**C4b**	Mm00437893_g1
**TLR2**	Mm00442346_m1
**TLR4**	Mm00445273_m1
**TLR2**	Mm00442346_m1
**Viperin/RSAD2**	Mm00491265_m1
**PUMA**	Mm00519268_m1
**β-actin**	Mm00607939_s1
**GAPDH**	Mm99999915_g1

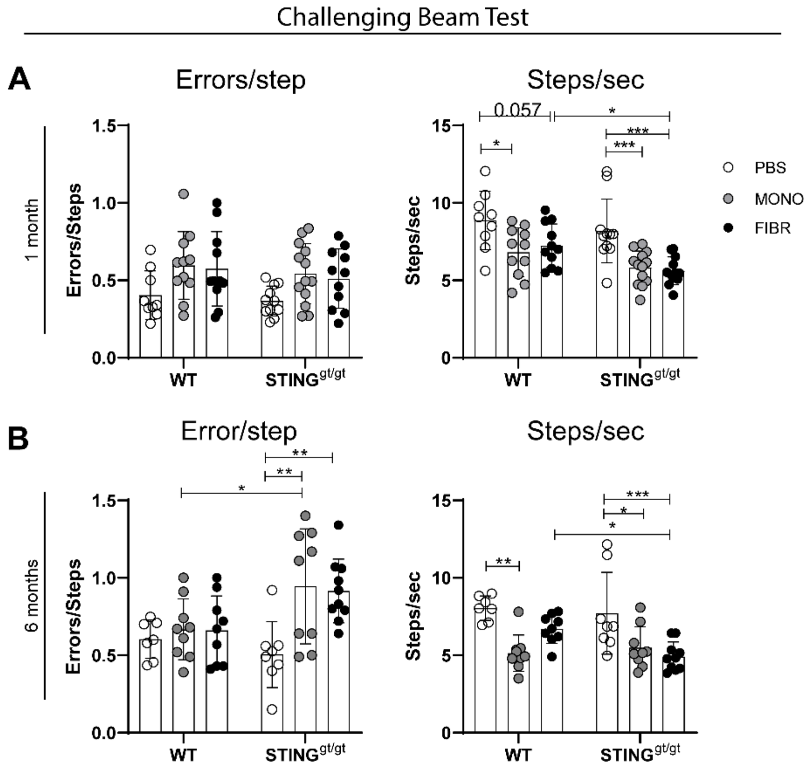

Supplementary figure 1: Challenging beam test. The challenging beam test was performed to evaluate motor behavior. At both 1-month **(A)** and 6-months post-injection **(B),** the number of errors/step and Steps/sec was calculated. n=7-10. For all graphs in the figure, a two-way ANOVA followed by Sidak's multiple comparisons was applied (test statistics in supplementary table 3). All values are mean +SD. $*<p=0.05$, $**p\leq0.01$, $***p\leq0.001$, $****p\leq0.0001$.

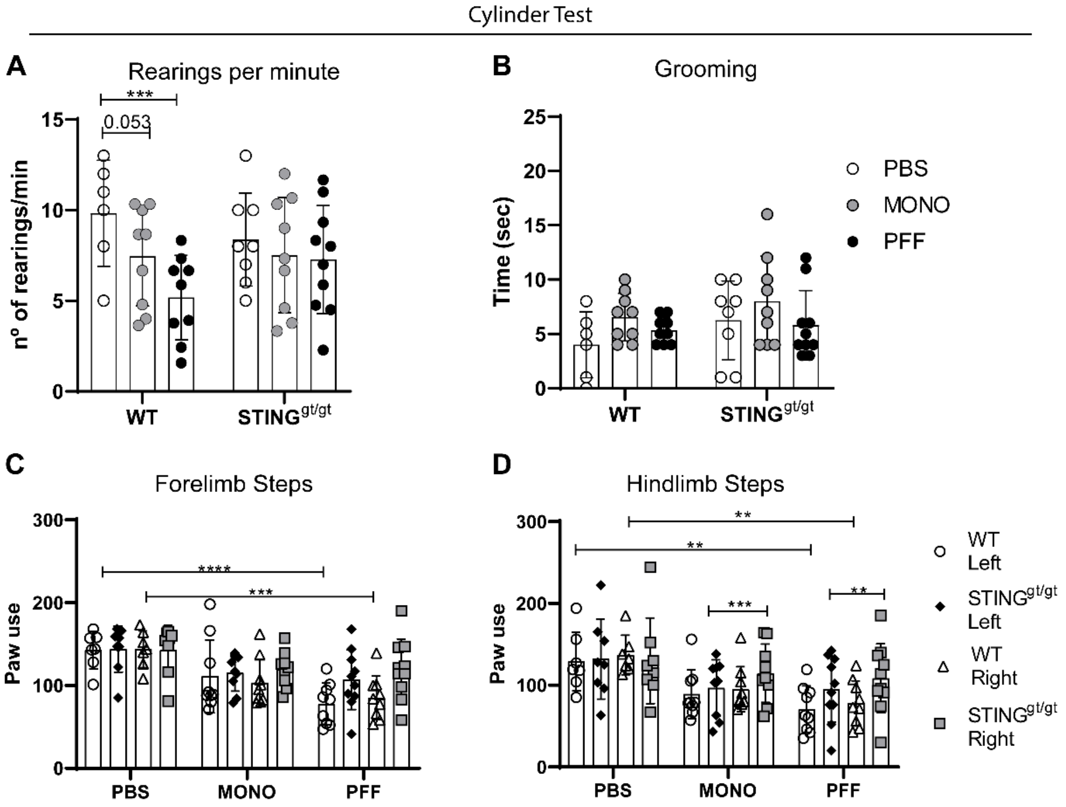

Supplementary figure 2: the Spontaneous activity test. At 6 months post-injection, the spontaneous activity in the cylinder test was performed to evaluate the motoric asymmetry. **A and B)** The number of rearings (A) and time spent grooming (B) were evaluated. **C and D)** The total paw use (steps) and the right and left forelimbs (C) and hindlimbs (D) were quantified. For A and B a two-way ANOVA followed by Sidak's multiple comparisons was applied (test statistics in supplementary table 3). For C and D). Three-way ANOVA with matched values followed by an uncorrected Fisher's LSD with Bonferroni correction applied. Corrected values are displayed on the graphs (*<p=0.05, **p≤0.01, ***p≤0.001, ****p≤0.0001). For all graphs, values are displayed as ± SD. n=6-10.

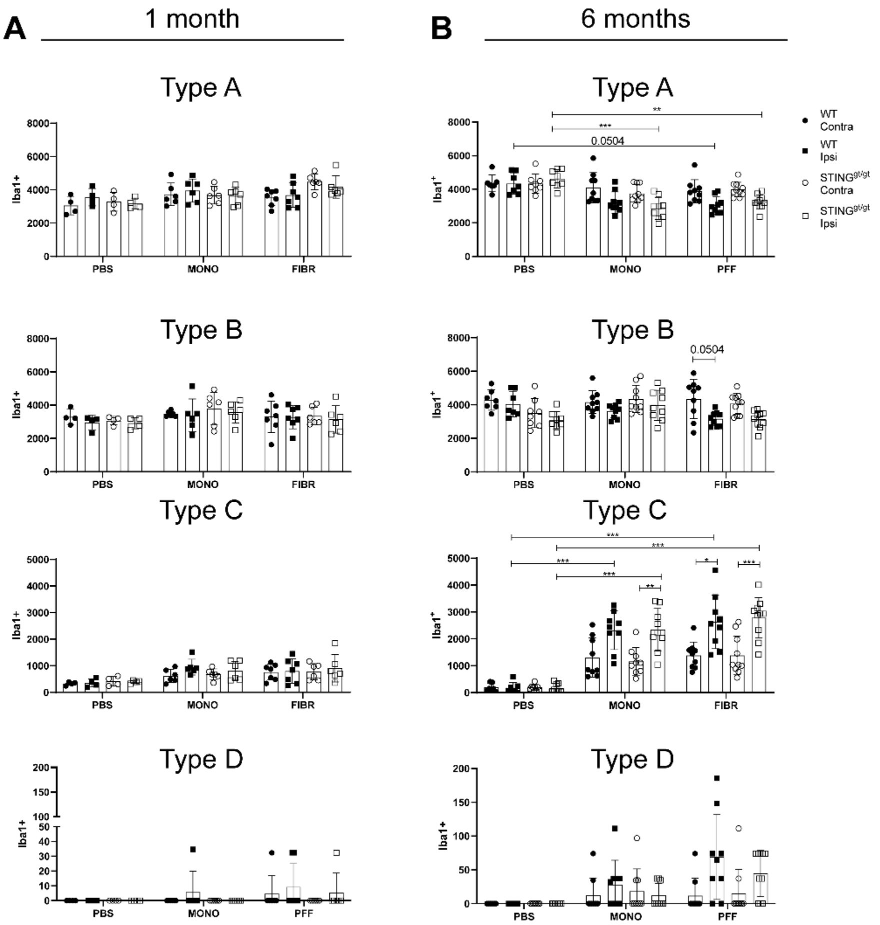

Supplementary figure 3: Stereological quantification of iba1 morphology reveals a long-term activation of microglia. A. A and C) Bar graphs illustrate the total number of either type A, B, C and D Iba1+ cells counted during the stereological quantification in the SN at 1 month p.i (B) and 6 months (C) post-injection . Three-way ANOVA with matched values followed by an uncorrected Fisher's LSD with Bonferroni correction applied. Corrected values are displayed on the graphs (*<p=0.05, **p≤0.01, ***p≤0.001, ****p≤0.0001). Data is displayed as ±SD, n=4-10. Note that total type D cells counted during the stereological quantification had a CE>0.01, thus no statistical test has been applied to the graphs displaying Type D Iba1^{+} cells.

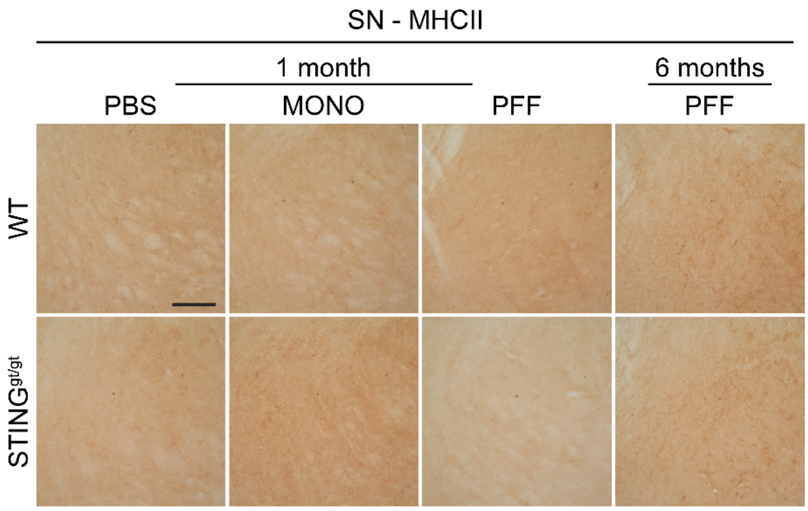

Supplementary Figure 4: Representative image of immune marker MHCII in the substantia nigra (SN) at 1 month and 6 months post-injection. No positive MHCII signaling was detected. Scalebar=100μm applies to all images.

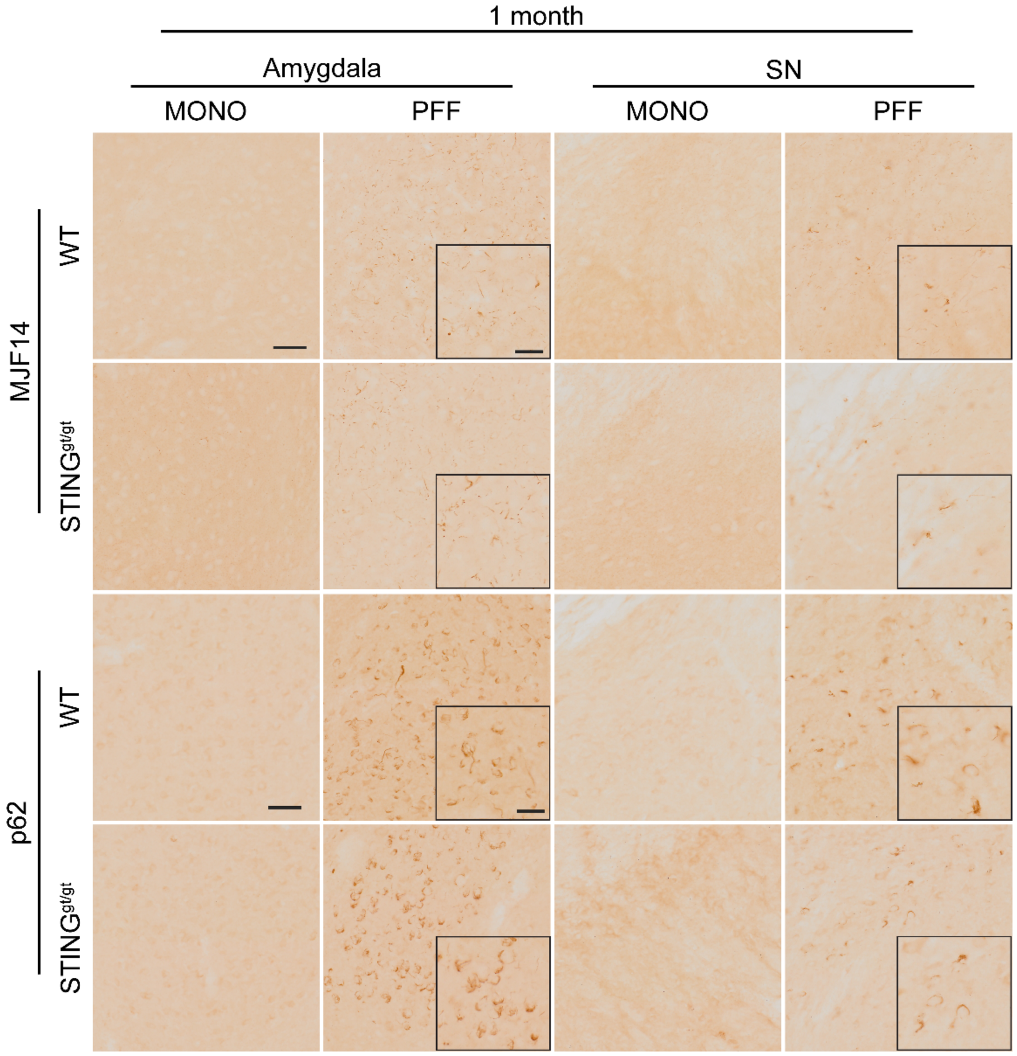

Supplementary figure 5: Photos of MJF14 and p62staining in amygdala and S. nigra at 1-month post-injection. As the area covered by p62 immunostaining in amygdala in PFF-mice was very variable (see main fig. 5), selected images show those animals with obvious p62 immuno-stained area (above average). Scalebar=50μm for all low magnification images and Scalebar=25μm for all inserts. One representative scalebar is shown for each area and marker.

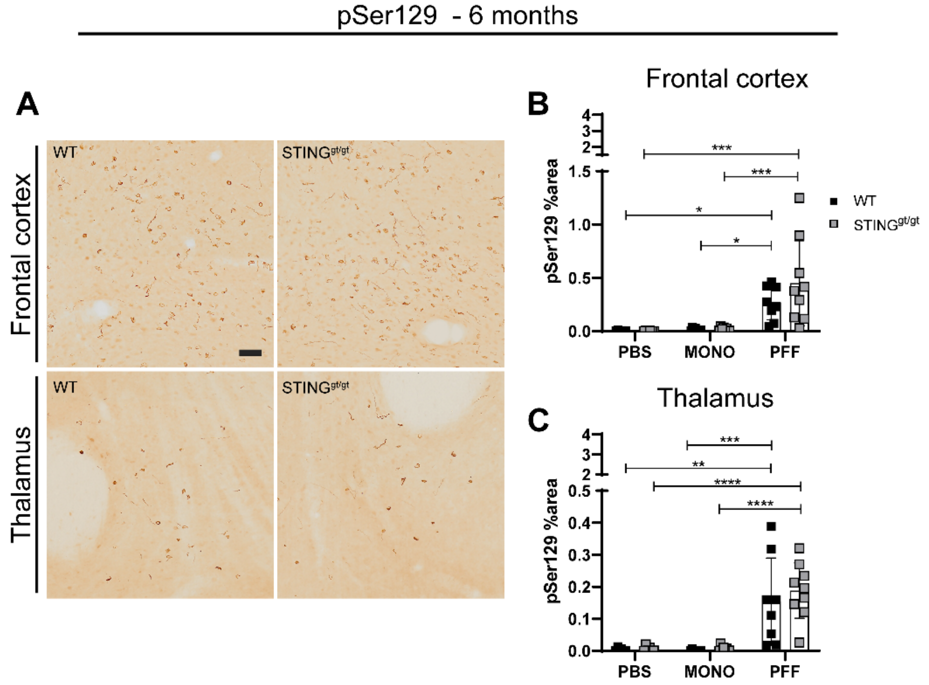

Supplementary figure 6: Injections of PFF lead to increase of pSer129-positive staining. **A)** Representative photos of pSer129 staining the frontal cortex and thalamus at 6 months post-injections. Scalebar=50μm applies to all images. **B, C)** Graphs show percentage of area covered by pser129 staining in PBS, MONO, and PFF-injected animals 6 months post-injected in the frontal cortex (B), and thalamus (C). Two-way ANOVA followed by Sidak's multiple comparisons (see further statistics in supplementary table 3). *<p=0.05, **p≤0.01, ***p≤0.001, ****p≤0.0001. All data are presented as mean ±SD. n= 6-10.

Supplementary table 3: 2-WAY ANOVA and 3-way ANOVA results

Figure 1- top panel

Challening beam Total time	ANOVA table	SS (Type III)	DF	MS	F (DFn, DFd)	P value
	Interaction	8.198	2	4.099	F (2, 61) = 0,6668	P=0,5170
	Genotype	25.83	1	25.83	F (1, 61) = 4,202	P=0,0447
	Treatment	145.3	2	72.63	F (2, 61) = 11,82	P<0,0001
	Residual	375	61	6.147		
**Challening beam Total steps**	**ANOVA table**	**SS (Type III)**	**DF**	**MS**	**F (DFn, DFd)**	**P value**
	Interaction	3.121	2	1.561	F (2, 61) = 1,544	P=0,2218
	Genotype	5.703	1	5.703	F (1, 61) = 5,642	P=0,0207
	Treatment	9.978	2	4.989	F (2, 61) = 4,935	P=0,0103
	Residual	61.66	61	1.011		
**Challening beam Total Errors**	**ANOVA table**	**SS (Type III)**	**DF**	**MS**	**F (DFn, DFd)**	**P value**
	Interaction	0.1243	2	0.06214	F (2, 60) = 0,0507:	P=0,9506
	Genotype	0.3984	1	0.3984	F (1, 60) = 0,3253	P=0,5706
	Treatment	17.28	2	8.641	F (2, 60) = 7,055	P=0,0018
	Residual	73.49	60	1.225		

Figure 1 - middle panel

Challening beam Total time	ANOVA table	SS (Type III)	DF	MS	F (DFn, DFd)	P value
	Interaction	34.34	2	17.17	F (2, 45) = 2,064	P=0,1388
	Genotype	17.42	1	17.42	F (1, 45) = 2,095	P=0,1547
	Treatment	211.4	2	105.7	F (2, 45) = 12,71	P<0,0001
	Residual	374.3	45	8.317		
**Challening beam Total steps**	**ANOVA table**	**SS (Type III)**	**DF**	**MS**	**F (DFn, DFd)**	**P value**
	Interaction	5.647	2	2.823	F (2, 46) = 1,499	P=0,2340
	Genotype	0.158	1	0.158	F (1, 46) = 0,08392	P=0,7734
	Treatment	16.71	2	8.354	F (2, 46) = 4,437	P=0,0173
	Residual	86.61	46	1.883		
**Challening beam Total Errors**	**ANOVA table**	**SS (Type III)**	**DF**	**MS**	**F (DFn, DFd)**	**P value**
	Interaction	14.85	2	7.427	F (2, 46) = 3,851	P=0,0284
	Genotype	7.767	1	7.767	F (1, 46) = 4,027	P=0,0507
	Treatment	16.15	2	8.077	F (2, 46) = 4,188	P=0,0213
	Residual	88.72	46	1.929		

Figure 1 – lower panel

Forelimbs Assymetry: %left paw/Right paw	ANOVA table	SS (Type III)	DF	MS	F (DFn, DFd)	P value
	Interaction	43.42	2	21.71	F (2, 46) = 0,4782	P=0,6230
	Genotype	8.512	1	8.512	F (1, 46) = 0,1875	P=0,6670
	Treatment	970.8	2	485.4	F (2, 46) = 10,69	P=0,0002
	Residual	2088	46	45.4		
**Hindlimb Assymetry: %Left paw/Right paw**	**ANOVA table**	**SS (Type III)**	**DF**	**MS**	**F (DFn, DFd)**	**P value**
	Interaction	817.5	2	408.7	F (2, 46) = 2,298	P=0,1119
	Genotype	14.16	1	14.16	F (1, 46) = 0,07963	P=0,7791
	Treatment	1035	2	517.6	F (2, 46) = 2,910	P=0,0646
	Residual	8182	46	177.9		

Figure 2C

Total Iba1 cells	ANOVA table	SS	DF	MS	F (DFn, DFd)	P value
	Treatment	22346221	2	11173111	F (2, 27) = 9,141	P=0,0009
	Genotype	665548	1	665548	F (1, 27) = 0,5445	P=0,4669
	Contra/Ipsi	1097	1	1097	F (1, 27) = 0,00327	P=0,9548
	Treatment x Genotype	2394906	2	1197453	F (2, 27) = 0,9797	P=0,3884
	Treatment x Contra/Ipsi	264208	2	132104	F (2, 27) = 0,3940	P=0,6782
	Genotype x Contra/Ipsi	1119722	1	1119722	F (1, 27) = 3,339	P=0,0787
	Treatment x Genotype x Contra/Ipsi	11113	2	5557	F (2, 27) = 0,01657	P=0,9836
	Subject	33001382	27	1222273		
	Residual	9053045	27	335298		

Figure 2D

% of Iba1 (WT PBS: Contra x Ipsi)	ANOVA table	SS	DF	MS	F (DFn, DFd)	P value	Repeate measure
	Hemisphere x Morphology	161.6	3	53.86	F (3, 12) = 5,557	P=0,0126	
	Hemisphere	0	1	0	F (1, 12) = 0,000	P>0,9999	
	Morphology	16311	3	5437	F (3, 12) = 3379	P<0,0001	
	Subject	19.31	12	1.609	F (12, 12) = 0,166(	P=0,9980	
	Residual	116.3	12	9.691			
**% of Iba1 (SITNG PBS: Contra x Ipsi)**	ANOVA table	SS	DF	MS	F (DFn, DFd)	P value	Repeate measure
	Hemisphere x Morphology	4.486	3	1.495	F (3, 12) = 0,1269	P=0,9423	
	Hemisphere	2.94	1	2.94	F (1, 12) = 0,2496	P=0,6264	
	Morphology	15385	3	5128	F (3, 12) = 764,3	P<0,0001	
	Subject	80.51	12	6.71	F (12, 12) = 0,5695	P=0,8287	
	Residual	141.4	12	11.78			
**% of Iba1 (WT MONO: Contra x Ipsi)**	ANOVA table	SS	DF	MS	F (DFn, DFd)	P value	Repeate measure
	Hemisphere x Cell morphology	95.46	3	31.82	F (3, 20) = 1,251	P=0,3179	
	Hemisphere	0.00003333	1	0.00003333	F (1, 20) = 1,310e-	P=0,9991	
	Cell morphology	20134	3	6711	F (3, 20) = 220,4	P<0,0001	
	Contra/Ipsi	609.1	20	30.45	F (20, 20) = 1,197	P=0,3457	
	Residual	508.8	20	25.44			
**% of Iba1 (WT PFF: Contra x Ipsi)**	**ANOVA table**	**SS**	**DF**	**MS**	**F (DFn, DFd)**	**P value**	Repeate measure
	Hemisphere x Cell morphology	2.614	3	0.8713	F (3, 24) = 0,0420(	P=0,9882	
	Hemisphere	7.143E-06	1	0.000007143	F (1, 24) = 3,448e-	P=0,9995	
	Cell morphology	23235	3	7745	F (3, 24) = 129,6	P<0,0001	
	Subject	1435	24	59.78	F (24, 24) = 2,886	P=0,0060	
	Residual	497.1	24	20.71			
**% of Iba1 (STING MONO: Contra x Ipsi)**	**ANOVA table**	**SS**	**DF**	**MS**	**F (DFn, DFd)**	**P value**	Repeate measure
	Hemisphere x Cell morphology	16.97	3	5.656	F (3, 20) = 0,2804	P=0,8389	
	Hemisphere	8.333E-06	1	0.000008333	F (1, 20) = 4,131e-	P=0,9995	
	Cell morphology	20471	3	6824	F (3, 20) = 151,5	P<0,0001	
	Contra/Ipsi	900.6	20	45.03	F (20, 20) = 2,232	P=0,0400	
	Residual	403.5	20	20.17			
**% of Iba1 (STING FFF: Contra x Ipsi)**	**ANOVA table**	**SS**	**DF**	**MS**	**F (DFn, DFd)**	**P value**	Repeate measure
	Hemisphere x Cell morphology	16.58	3	5.527	F (3, 20) = 0,3408	P=0,7961	
	Hemisphere	2.083E-06	1	0.000002083	F (1, 20) = 1,285e-	P=0,9997	
	Cell morphology	21000	3	7000	F (3, 20) = 177,2	P<0,0001	
	Contra/Ipsi	790	20	39.5	F (20, 20) = 2,436	P=0,0265	
	Residual	324.3	20	16.22			
**% of Iba1 (Ipsi PFF: WT x STING)**	**ANOVA table**	**SS**	**DF**	**MS**	**F (DFn, DFd)**	**P value**	
	Interaction	149.9	3	49.96	F (3, 44) = 1,546	P=0,2159	
	Genotype	3.432	1	3.432	F (1, 44) = 0,1062	P=0,7460	
	Morphology	20795	3	6932	F (3, 44) = 214,6	P<0,0001	
	Residual	1421	44	32.31			
**% of Iba1 (Ipsi MONO: WT x STING)**	**ANOVA table**	**SS**	**DF**	**MS**	**F (DFn, DFd)**	**P value**	
	Interaction	74.49	3	24.83	F (3, 40) = 0,6916	P=0,5626	
	Genotype	8.333E-06	1	0.000008333	F (1, 40) = 2,321e-	P=0,9996	
	Morphology	19192	3	6397	F (3, 40) = 178,2	P<0,0001	
	Residual	1436	40	35.91			
**% of Iba1 (Contra PFF: WT x STING)**	**ANOVA table**	**SS**	**DF**	**MS**	**F (DFn, DFd)**	**P value**	
	Interaction	132.2	3	44.08	F (3, 44) = 1,290	P=0,2896	
	Genotype	7.738E-06	1	0.000007738	F (1, 44) = 2,265e-	P=0,9996	
	Morphology	22421	3	7474	F (3, 44) = 218,8	P<0,0001	
	Residual	1503	44	34.16			
**% of Iba1 (Contra MONO: WT x STING)**	**ANOVA table**	**SS**	**DF**	**MS**	**F (DFn, DFd)**	**P value**	
	Interaction	14.34	3	4.78	F (3, 40) = 0,1940	P=0,8999	
	Genotype	0	1	0	F (1, 40) = 0,000	P>0,9999	
	Morphology	21436	3	7145	F (3, 40) = 289,9	P<0,0001	
	Residual	985.8	40	24.64			
**%Iba1 (Contra PBS: WT x STING)**	**ANOVA table**	**SS**	**DF**	**MS**	**F (DFn, DFd)**	**P value**	
	Interaction	54.74	3	18.25	F (3, 24) = 1,790	P=0,1759	
	Genotype	0.1985	1	0.1985	F (1, 24) = 0,01947	P=0,8902	
	Morphology	16059	3	5353	F (3, 24) = 525,3	P<0,0001	
	Residual	244.6	24	10.19			
**%Iba1 (Ipsi PBS: WT x STING)**	**ANOVA table**	**SS**	**DF**	**MS**	**F (DFn, DFd)**	**P value**	
	Interaction	52.28	3	17.43	F (3, 24) = 3,704	P=0,0254	
	Genotype	1.611	1	1.611	F (1, 24) = 0,3424	P=0,5639	
	Morphology	15697	3	5232	F (3, 24) = 1112	P<0,0001	
	Residual	112.9	24	4.705			

Figure 2E

**Total Iba1 cells**	**ANOVA table**	**SS**	**DF**	**MS**	**F (DFn, DFd)**	**P value**
	Treatment	98452	1	98452	F (1, 33) = 0,1202	P=0,7310
	Genotype	9047	1	9047	F (1, 33) = 0,01105	P=0,9169
	contra/ipsi	1557671	1	#####	F (1, 33) = 3,013	P=0,0919
	Treatment x Genotype	535229	1	#####	F (1, 33) = 0,6536	P=0,4246
	Treatment x contra/ipsi	47351	1	47351	F (1, 33) = 0,09159	P=0,7641
	Genotype x contra/ipsi	294399	1	#####	F (1, 33) = 0,5694	P=0,4558
	Treatment x Genotype x contra/ipsi	1973	1	1973	F (1, 33) = 0,00381(	P=0,9511
	Subject	27023093	33	#####		
	Residual	17061144	33	#####		

Figure 2F

% of Iba1 (WT PBS: Contra x Ipsi)	ANOVA table	SS	DF	MS	F (DFn, DFd)	P value	Repeate measure
	Hemisphere x Morphology	13.64	3	4.548	F (3, 24) = 0,3083	P=0,8191	
	Hemisphere	0.00002857	1	3E-05	F (1, 24) = 1,937e-0	P=0,9989	
	Morphology	32245	3	10748	F (3, 24) = 493,9	P<0,0001	
	Subject	522.3	24	21.76	F (24, 24) = 1,475	P=0,1737	
	Residual	354	24	14.75			
**% of Iba1 (STING PBS: Contra x Ipsi)**	ANOVA table	SS	DF	MS	F (DFn, DFd)	P value	Repeate measure
	Hemisphere x Morphology	167.5	3	55.85	F (3, 28) = 5,824	P=0,0032	
	Hemisphere	2.827	1	2.827	F (1, 28) = 0,2947	P=0,5915	
	Morphology	39042	3	13014	F (3, 28) = 284,7	P<0,0001	
	Subject	1280	28	45.71	F (28, 28) = 4,767	P<0,0001	
	Residual	268.5	28	9.59			
**% of Iba1 (WT MONO: Contra x Ipsi)**	ANOVA table	SS	DF	MS	F (DFn, DFd)	P value	Repeate measure
	Hemisphere x Cell morphology	695.2	3	231.7	F (3, 32) = 14,82	P<0,0001	
	Hemisphere	0.06722	1	0.067	F (1, 32) = 0,004299	P=0,9481	
	Cell morphology	20054	3	6685	F (3, 32) = 120,0	P<0,0001	
	contra/ipsi	1782	32	55.69	F (32, 32) = 3,561	P=0,0003	
	Residual	500.4	32	15.64			
**% of Iba1 (WT PFF: Contra x Ipsi)**	**ANOVA table**	**SS**	**DF**	**MS**	**F (DFn, DFd)**	**P value**	Repeate measure
	Hemisphere x Cell morphology	1575	3	525	F (3, 32) = 19,37	P<0,0001	
	Hemisphere	8.208	1	8.208	F (1, 32) = 0,3028	P=0,5859	
	Cell morphology	18490	3	6163	F (3, 32) = 111,5	P<0,0001	
	Contra/Ipsi	1769	32	55.27	F (32, 32) = 2,039	P=0,0239	
	Residual	867.3	32	27.1			
**% of Iba1 (STING MONO: Contra x Ipsi)**	**ANOVA table**	**SS**	**DF**	**MS**	**F (DFn, DFd)**	**P value**	Repeate measure
	Hemisphere x Cell morphology	1267	3	422.2	F (3, 32) = 26,55	P<0,0001	
	Hemisphere	5.556E-06	1	6E-06	F (1, 32) = 3,493e-0	P=0,9995	
	Cell morphology	20860	3	6953	F (3, 32) = 146,9	P<0,0001	
	Contra/Ipsi	1515	32	47.34	F (32, 32) = 2,977	P=0,0014	
	Residual	508.9	32	15.9			
**% of Iba1 (STING FFF: Contra x Ipsi)**	**ANOVA table**	**SS**	**DF**	**MS**	**F (DFn, DFd)**	**P value**	Repeate measure
	Hemisphere x Cell morphology	1815	3	605	F (3, 36) = 34,04	P<0,0001	
	Hemisphere	2.019E-27	1	2E-27	F (1, 36) = 1,136e-0	P>0,9999	
	Cell morphology	19829	3	6610	F (3, 36) = 193,2	P<0,0001	
	Contra/Ipsi	1232	36	34.21	F (36, 36) = 1,925	P=0,0265	
	Residual	639.8	36	17.77			
**% of Iba1 (Ipsi PFF: WT x STING)**	**ANOVA table**	**SS**	**DF**	**MS**	**F (DFn, DFd)**	**P value**	
	ANOVA table	SS (Type III)	DF	MS	F (DFn, DFd)	P value	
	Interaction	20.67	3	6.889	F (3, 68) = 0,2137	P=0,8866	
	Genotype	1.345E-28	1	1E-28	F (1, 68) = 4,173e-0	P>0,9999	
	Cell morphology	15369	3	5123	F (3, 68) = 158,9	P<0,0001	
**% of Iba1 (Ipsi MONO: WT x STING)**	**ANOVA table**	**SS**	**DF**	**MS**	**F (DFn, DFd)**	**P value**	
	Interaction	98.48	3	32.83	F (3, 64) = 0,9276	P=0,4326	
	Genotype	1.291	1	1.291	F (1, 64) = 0,03647	P=0,8492	
	Cell morphology	17303	3	5768	F (3, 64) = 163,0	P<0,0001	
	Residual	2265	64	35.39			
**% of Iba1 (Contra PFF: WT x STING)**	**ANOVA table**	**SS**	**DF**	**MS**	**F (DFn, DFd)**	**P value**	
	Interaction	33.25	3	11.08	F (3, 68) = 0,3255	P=0,8069	
	Genotype	8.64	1	8.64	F (1, 68) = 0,2538	P=0,6160	
	Cell morphology	26203	3	8734	F (3, 68) = 256,6	P<0,0001	
	Residual	2315	68	34.04			
**% of Iba1 (Contra MONO: WT x STING)**	**ANOVA table**	**SS**	**DF**	**MS**	**F (DFn, DFd)**	**P value**	
	Interaction	122.6	3	40.86	F (3, 64) = 1,281	P=0,2885	
	Genotype	1.954	1	1.954	F (1, 64) = 0,06124	P=0,8053	
	Cell morphology	25351	3	8450	F (3, 64) = 264,9	P<0,0001	
	Residual	2042	64	31.9			
**%Iba1 (Contra PBS: WT x STING)**	**ANOVA table**	**SS**	**DF**	**MS**	**F (DFn, DFd)**	**P value**	
	Interaction	190.3	3	63.43	F (3, 52) = 2,997	P=0,0390	
	Genotype	1.071E-06	1	1E-06	F (1, 52) = 5,062e-0	P=0,9998	
	Morphologt	34331	3	11444	F (3, 52) = 540,7	P<0,0001	
	Residual	1101	52	21.17			
**%Iba1 (Ipsi PBS: WT x STING)**	**ANOVA table**	**SS**	**DF**	**MS**	**F (DFn, DFd)**	**P value**	
	Interaction	500	3	166.7	F (3, 52) = 6,545	P=0,0008	
	Genotype	2.66	1	2.66	F (1, 52) = 0,1044	P=0,7479	
	Morphology	35984	3	11995	F (3, 52) = 471,0	P<0,0001	
	Residual	1324	52	25.46			

Figure 3B

MHCII Striatum Average ramified MHCII	ANOVA table	SS (Type III)	DF	MS	F (DFn, DFd)	P value
	Interaction	55,4	1	55,4	F (1, 20) = 0,04929	P=0,8266
	Genotype	32,96	1	32,96	F (1, 20) = 0,02932	P=0,8658
	Treatment	14562	1	14562	F (1, 20) = 12,96	P=0,0018
	Residual	22481	20	1124		

Figure 4B

CD68 striatum %area	ANOVA table	SS (Type III)	DF	MS	F (DFn, DFd)	P value
	Interaction	4,474	2	2,237	F (2, 28) = 9,942	P=0,0005
	Treatment	6,999	2	3,499	F (2, 28) = 15,55	P<0,0001
	Genotype	1,411	1	1,411	F (1, 28) = 6,270	P=0,0184
	Residual	6,3	28	0,225		

Figure 4C

CD68 striatum %area	ANOVA table	SS (Type III)	DF	MS	F (DFn, DFd)	P value
	Interaction	0,09374	2	0,047	F (2, 46) = 2,234	P=0,1186
	Treatment	3,431	2	1,715	F (2, 46) = 81,77	P<0,0001
	Genotype	0,0001341	1	1E-04	F (1, 46) = 0,006392	P=0,9366
	Residual	0,965	46	0,021		

Figure 4E

CD68 SN %area	ANOVA table	SS (Type III)	DF	MS	F (DFn, DFd)	P value
	Interaction	0.3017	2	0.1509	F (2, 28) = 0,9934	P=0,3830
	Treatment	0.2675	2	0.1338	F (2, 28) = 0,8808	P=0,4256
	Genotype	0.2061	1	0.2061	F (1, 28) = 1,357	P=0,2539
	Residual	4.252	28	0.1519		

Figure 4F

CD68 SN %area	ANOVA table	SS (Type III)	DF	MS	F (DFn, DFd)	P value
	Interaction	0.2582	2	0.1291	F (2, 35) = 1,486	P=0,2402
	Treatment	1.768	2	0.8839	F (2, 35) = 10,17	P=0,0003
	Genotype	0.0124	1	0.0124	F (1, 35) = 0,1427	P=0,7079
	Residual	3.041	35	0.08689		

Figure 5A Prefrontal cortex

TNF-a	ANOVA table	SS (Type III)	DF	MS	F (DFn, DFd)	P value
	Interaction	1.093E-07	1	1.093E-07	F (1, 11) = 2,105	P=0,1747
	Genotype	9.405E-08	1	9.405E-08	F (1, 11) = 1,812	P=0,2054
	Treatment	7.855E-08	1	7.855E-08	F (1, 11) = 1,513	P=0,2443
	Residual	5.71E-07	11	5.191E-08		
**IFN-b**	**ANOVA table**	**SS (Type III)**	**DF**	**MS**	**F (DFn, DFd)**	**P value**
	Interaction	4.15E-09	1	4.15E-09	F (1, 12) = 2,860	P=0,1166
	Genotype	1.382E-11	1	1.382E-11	F (1, 12) = 0,00952	P=0,9239
	Treatment	6.781E-10	1	6.781E-10	F (1, 12) = 0,4673	P=0,5072
	Residual	1.741E-08	12	1.451E-09		
**IL1-b**	**ANOVA table**	**SS (Type III)**	**DF**	**MS**	**F (DFn, DFd)**	**P value**
	Interaction	7.744E-11	1	7.744E-11	F (1, 13) = 0,1450	P=0,7095
	Genotype	3.6E-11	1	3.6E-11	F (1, 13) = 0,06740	P=0,7992
	Treatment	1.318E-09	1	1.318E-09	F (1, 13) = 2,467	P=0,1403
	Residual	6.944E-09	13	5.341E-10		
**IL-6**	**ANOVA table**	**SS (Type III)**	**DF**	**MS**	**F (DFn, DFd)**	**P value**
	Interaction	4.277E-10	1	4.277E-10	F (1, 16) = 1,107	P=0,3084
	Genotype	1.632E-09	1	1.632E-09	F (1, 16) = 4,225	P=0,0565
	Treatment	2.9E-10	1	2.9E-10	F (1, 16) = 0,7505	P=0,3991
	Residual	6.182E-09	16	3.864E-10		
**CXCL10**	**ANOVA table**	**SS (Type III)**	**DF**	**MS**	**F (DFn, DFd)**	**P value**
	Interaction	8.085E-08	1	8.085E-08	F (1, 15) = 1,174	P=0,2958
	Genotype	2.31E-09	1	2.31E-09	F (1, 15) = 0,03353	P=0,8572
	Treatment	2.254E-07	1	2.254E-07	F (1, 15) = 3,272	P=0,0906
	Residual	1.033E-06	15	6.89E-08		
**Mx1**	**ANOVA table**	**SS (Type III)**	**DF**	**MS**	**F (DFn, DFd)**	**P value**
	Interaction	2.287E-09	1	2.287E-09	F (1, 15) = 2,564	P=0,1302
	Genotype	1.849E-09	1	1.849E-09	F (1, 15) = 2,073	P=0,1704
	Treatment	2.808E-11	1	2.808E-11	F (1, 15) = 0,03149	P=0,8615
	Residual	1.338E-08	15	8.919E-10		
**Viperin**	**ANOVA table**	**SS (Type III)**	**DF**	**MS**	**F (DFn, DFd)**	**P value**
	Interaction	7.947E-10	1	7.947E-10	F (1, 17) = 0,05542	P=0,8167
	Genotype	1.756E-10	1	1.756E-10	F (1, 17) = 0,01225	P=0,9132
	Treatment	1.905E-08	1	1.905E-08	F (1, 17) = 1,328	P=0,2651
	Residual	2.438E-07	17	1.434E-08		
**CCL2**	**ANOVA table**	**SS (Type III)**	**DF**	**MS**	**F (DFn, DFd)**	**P value**
	Interaction	1.064E-09	1	1.064E-09	F (1, 14) = 3,758	P=0,0730
	Genotype	1.321E-09	1	1.321E-09	F (1, 14) = 4,667	P=0,0486
	Treatment	6.847E-12	1	6.847E-12	F (1, 14) = 0,02419	P=0,8786
	Residual	3.963E-09	14	2.831E-10		
**TLR4**	**ANOVA table**	**SS (Type III)**	**DF**	**MS**	**F (DFn, DFd)**	**P value**
	Interaction	3.016E-09	1	3.016E-09	F (1, 15) = 12,43	P=0,0031
	Genotype	2.311E-11	1	2.311E-11	F (1, 15) = 0,09527	P=0,7618
	Treamtent	1.29E-10	1	1.29E-10	F (1, 15) = 0,5320	P=0,4770
	Residual	3.638E-09	15	2.425E-10		
**TLR2**	**ANOVA table**	**SS (Type III)**	**DF**	**MS**	**F (DFn, DFd)**	**P value**
	Interaction	8.684E-09	1	8.684E-09	F (1, 15) = 9,633	P=0,0073
	Row Factor	3.276E-09	1	3.276E-09	F (1, 15) = 3,634	P=0,0760
	Column Factor	1.636E-08	1	1.636E-08	F (1, 15) = 18,15	P=0,0007
	Residual	1.352E-08	15	9.014E-10		
**C1q**	**ANOVA table**	**SS (Type III)**	**DF**	**MS**	**F (DFn, DFd)**	**P value**
	Interaction	0.00000266	1	0.00000266	F (1, 16) = 0,1650	P=0,6900
	Genotype	0.00001058	1	0.00001058	F (1, 16) = 0,6561	P=0,4298
	Treatment	0.0001935	1	0.0001935	F (1, 16) = 12,00	P=0,0032
	Residual	0.0002579	16	0.00001612		
**C4b**	**ANOVA table**	**SS (Type III)**	**DF**	**MS**	**F (DFn, DFd)**	**P value**
	Interaction	0.00002439	1	0.00002439	F (1, 17) = 1,605	P=0,2223
	Genotype	2.376E-08	1	2.376E-08	F (1, 17) = 0,00156	P=0,9689
	Treatment	0.00001176	1	0.00001176	F (1, 17) = 0,7742	P=0,3912
	Residual	0.0002583	17	0.00001519		
**PUMA**	**ANOVA table**	**SS (Type III)**	**DF**	**MS**	**F (DFn, DFd)**	**P value**
	Interaction	4.92E-07	1	0.000000492	F (1, 17) = 0,9161	P=0,3519
	Genotype	6.423E-07	1	6.423E-07	F (1, 17) = 1,196	P=0,2894
	Treatment	1.959E-06	1	0.000001959	F (1, 17) = 3,647	P=0,0732
	Residual	0.00000913	17	5.371E-07		
**TREM2**	**ANOVA table**	**SS (Type III)**	**DF**	**MS**	**F (DFn, DFd)**	**P value**
	Interaction	2.897E-07	1	2.897E-07	F (1, 15) = 1,303	P=0,2716
	Genotype	1.314E-07	1	1.314E-07	F (1, 15) = 0,5907	P=0,4541
	Treatment	1.109E-06	1	0.000001109	F (1, 15) = 4,988	P=0,0412
	Residual	3.336E-06	15	2.224E-07		

Figure 5B Striatum

TNF-a	ANOVA table	SS (Type III)	DF	MS	F (DFn	P value
	Interaction	3.535E-11	1	0	F (1, 1	P=0,5490
	Genotype	2.336E-10	1	0	F (1, 1	P=0,1350
	Treatment	1.319E-10	1	0	F (1, 1	P=0,2541
	Residual	1.509E-09	16	0		
IFN-b	ANOVA table	SS (Type III)	DF	MS	F (DFn	P value
	Interaction	4.93E-09	1	0	F (1, 1	P=0,0513
	Genotype	1.532E-12	1	0	F (1, 1	P=0,9708
	Treatment	7.58E-10	1	0	F (1, 1	P=0,4210
	Residual	1.778E-08	16	0		
IL1-b	ANOVA table	SS (Type III)	DF	MS	F (DFn	P value
	Interaction	1.283E-09	1	0	F (1, 1	P=0,0994
	Genotype	2.544E-09	1	0	F (1, 1	P=0,0252
	Treatment	5.573E-09	1	0	F (1, 1	P=0,0021
	Residual	7.18E-09	17	0		
IL-6	ANOVA table	SS (Type III)	DF	MS	F (DFn	P value
	Interaction	7.412E-10	1	0	F (1, 1	P=0,0835
	Genotype	3.249E-10	1	0	F (1, 1	P=0,2402
	Treatment	2.335E-10	1	0	F (1, 1	P=0,3167
	Residual	3.728E-09	17	0		
CXCL10	ANOVA table	SS (Type III)	DF	MS	F (DFn	P value
	Interaction	4.19E-08	1	0	F (1, 1	P=0,5118
	Genotype	8.986E-07	1	0	F (1, 1	P=0,0065
	Treatment	3.262E-07	1	0	F (1, 1	P=0,0789
	Residual	0.000001587	17	0		
Mx1	ANOVA table	SS (Type III)	DF	MS	F (DFn	P value
	Interaction	2.452E-10	1	0	F (1, 1	P=0,5431
	Genotype	6.602E-10	1	0	F (1, 1	P=0,3228
	Treatment	3.83E-09	1	0	F (1, 1	P=0,0253
	Residual	1.082E-08	17	0		
Viperin	ANOVA table	SS (Type III)	DF	MS	F (DFn	P value
	Interaction	9.011E-12	1	0	F (1, 1	P=0,9786
	Genotype	5.59E-08	1	0	F (1, 1	P=0,0471
	Treatment	2.689E-11	1	0	F (1, 1	P=0,9631
	Residual	2.074E-07	17	0		
CCL2	ANOVA table	SS (Type III)	DF	MS	F (DFn	P value
	Interaction	7.652E-11	1	0	F (1, 1	P=0,7321
	Genotype	4.851E-09	1	0	F (1, 1	P=0,0131
	Treatment	1.422E-09	1	0	F (1, 1	P=0,1519
	Residual	1.074E-08	17	0		
TLR4	ANOVA table	SS (Type III)	DF	MS	F (DFn	P value
	Interaction	1.778E-10	1	0	F (1, 1	P=0,3747
	Genotype	5.75E-10	1	0	F (1, 1	P=0,1195
	Treamtent	3.377E-10	1	0	F (1, 1	P=0,2261
	Residual	3.638E-09	17	0		
TLR2	ANOVA table	SS (Type III)	DF	MS	F (DFn	P value
	Interaction	2.003E-08	1	0	F (1, 1	P=0,0735
	Row Factor	1.837E-08	1	0	F (1, 1	P=0,0853
	Column Factor	1.494E-07	1	0	F (1, 1	P<0,0001
	Residual	9.361E-08	17	0		
C1q	ANOVA table	SS (Type III)	DF	MS	F (DFn	P value
	Interaction	0.00001179	1	0	F (1, 1	P=0,7155
	Genotype	0.0003339	1	0	F (1, 1	P=0,0651
	Treatment	0.001095	1	0	F (1, 1	P=0,0024
	Residual	0.00146	17	0		
C4b	ANOVA table	SS (Type III)	DF	MS	F (DFn	P value
	Interaction	0.0001736	1	0	F (1, 1	P=0,3216
	Genotype	0.0005695	1	0	F (1, 1	P=0,0819
	Treatment	0.002379	1	0	F (1, 1	P=0,0015
	Residual	0.002831	17	0		
PUMA	ANOVA table	SS (Type III)	DF	MS	F (DFn	P value
	Interaction	5.224E-07	1	0	F (1, 1	P=0,3134
	Genotype	0.0000011	1	0	F (1, 1	P=0,1500
	Treatment	0.000002578	1	0	F (1, 1	P=0,0339
	Residual	0.000008229	17	0		
TREM2	ANOVA table	SS (Type III)	DF	MS	F (DFn	P value
	Interaction	0.000002837	1	0	F (1, 1	P=0,2837
	Genotype	0.00001182	1	0	F (1, 1	P=0,0373
	Treatment	0.00001802	1	0	F (1, 1	P=0,0126
	Residual	0.00003936	17	0		

Figure 5C Ventral midbrain

TNF-a	ANOVA table	SS (Type III)	DF	MS	F (DFn, DFd)	P value
	Interaction	3.919E-10	1	3.919E-10	F (1, 15) = 1,170	P=0,2965
	Genotype	1.312E-10	1	1.312E-10	F (1, 15) = 0,3918	P=0,5408
	Treatment	2.939E-10	1	2.939E-10	F (1, 15) = 0,8773	P=0,3638
	Residual	5.024E-09	15	3.349E-10		
**IFN-b**	**ANOVA table**	**SS (Type III)**	**DF**	**MS**	**F (DFn, DFd)**	**P value**
	Interaction	2.736E-09	1	2.736E-09	F (1, 13) = 5,395	P=0,0371
	Genotype	3.444E-11	1	3.444E-11	F (1, 13) = 0,06791	P=0,7985
	Treatment	2.249E-12	1	2.249E-12	F (1, 13) = 0,004435	P=0,9479
	Residual	6.593E-09	13	5.072E-10		
**IL1-b**	**ANOVA table**	**SS (Type III)**	**DF**	**MS**	**F (DFn, DFd)**	**P value**
	Interaction	1.599E-09	1	1.599E-09	F (1, 18) = 1,764	P=0,2007
	Genotype	1.532E-09	1	1.532E-09	F (1, 18) = 1,691	P=0,2099
	Treatment	1.187E-09	1	1.187E-09	F (1, 18) = 1,309	P=0,2675
	Residual	1.631E-08	18	9.062E-10		
**IL-6**	**ANOVA table**	**SS (Type III)**	**DF**	**MS**	**F (DFn, DFd)**	**P value**
	Interaction	2.878E-10	1	2.878E-10	F (1, 18) = 1,272	P=0,2742
	Genotype	1.089E-09	1	1.089E-09	F (1, 18) = 4,814	P=0,0416
	Treatment	4.105E-10	1	4.105E-10	F (1, 18) = 1,814	P=0,1947
	Residual	4.073E-09	18	2.263E-10		
**CXCL10**	**ANOVA table**	**SS (Type III)**	**DF**	**MS**	**F (DFn, DFd)**	**P value**
	Interaction	2.242E-08	1	2.242E-08	F (1, 17) = 1,018	P=0,3272
	Genotype	1.643E-07	1	1.643E-07	F (1, 17) = 7,459	P=0,0142
	Treatment	1.913E-09	1	1.913E-09	F (1, 17) = 0,08687	P=0,7718
	Residual	3.744E-07	17	2.202E-08		
**Mx1**	**ANOVA table**	**SS (Type III)**	**DF**	**MS**	**F (DFn, DFd)**	**P value**
	Interaction	4.104E-10	1	4.104E-10	F (1, 18) = 0,6564	P=0,4284
	Genotype	7.731E-15	1	7.731E-15	F (1, 18) = 1,236e-005	P=0,9972
	Treatment	2.013E-09	1	2.013E-09	F (1, 18) = 3,220	P=0,0895
	Residual	1.125E-08	18	6.252E-10		
**Viperin**	**ANOVA table**	**SS (Type III)**	**DF**	**MS**	**F (DFn, DFd)**	**P value**
	Interaction	2.123E-08	1	2.123E-08	F (1, 18) = 3,096	P=0,0955
	Genotype	7.752E-08	1	7.752E-08	F (1, 18) = 11,31	P=0,0035
	Treatment	2.504E-08	1	2.504E-08	F (1, 18) = 3,652	P=0,0721
	Residual	1.234E-07	18	6.856E-09		
**CCL2**	**ANOVA table**	**SS (Type III)**	**DF**	**MS**	**F (DFn, DFd)**	**P value**
	Interaction	2.639E-09	1	2.639E-09	F (1, 18) = 1,167	P=0,2943
	Genotype	4.603E-10	1	4.603E-10	F (1, 18) = 0,2035	P=0,6573
	Treatment	5.832E-11	1	5.832E-11	F (1, 18) = 0,02579	P=0,8742
	Residual	4.071E-08	18	2.262E-09		
**TLR4**	**ANOVA table**	**SS (Type III)**	**DF**	**MS**	**F (DFn, DFd)**	**P value**
	Interaction	5.092E-10	1	5.092E-10	F (1, 17) = 1,264	P=0,2765
	Genotype	7.406E-10	1	7.406E-10	F (1, 17) = 1,839	P=0,1928
	Treamtent	4.062E-10	1	4.062E-10	F (1, 17) = 1,009	P=0,3293
	Residual	6.846E-09	17	4.027E-10		
**TLR2**	**ANOVA table**	**SS (Type III)**	**DF**	**MS**	**F (DFn, DFd)**	**P value**
	Interaction	3.79E-10	1	3.79E-10	F (1, 17) = 0,2847	P=0,6005
	Row Factor	5.133E-09	1	5.133E-09	F (1, 17) = 3,856	P=0,0661
	Column Factor	4.174E-10	1	4.174E-10	F (1, 17) = 0,3136	P=0,5828
	Residual	2.263E-08	17	1.331E-09		
**C1q**	**ANOVA table**	**SS (Type III)**	**DF**	**MS**	**F (DFn, DFd)**	**P value**
	Interaction	0.00005987	1	0.00005987	F (1, 17) = 5,770	P=0,0280
	Genotype	0.000202	1	0.000202	F (1, 17) = 19,47	P=0,0004
	Treatment	0.00007299	1	0.00007299	F (1, 17) = 7,034	P=0,0168
	Residual	0.0001764	17	0.00001038		
**C4b**	**ANOVA table**	**SS (Type III)**	**DF**	**MS**	**F (DFn, DFd)**	**P value**
	Interaction	0.00005984	1	0.00005984	F (1, 17) = 2,139	P=0,1619
	Genotype	0.000126	1	0.000126	F (1, 17) = 4,504	P=0,0488
	Treatment	0.00007249	1	0.00007249	F (1, 17) = 2,591	P=0,1259
	Residual	0.0004756	17	0.00002798		
**PUMA**	**ANOVA table**	**SS (Type III)**	**DF**	**MS**	**F (DFn, DFd)**	**P value**
	Interaction	0.000009358	1	0.000009358	F (1, 18) = 3,582	P=0,0746
	Genotype	0.000005413	1	0.000005413	F (1, 18) = 2,072	P=0,1672
	Treatment	0.000004897	1	0.000004897	F (1, 18) = 1,874	P=0,1878
	Residual	0.00004702	18	0.000002612		
**TREM2**	**ANOVA table**	**SS (Type III)**	**DF**	**MS**	**F (DFn, DFd)**	**P value**
	Interaction	0.000001649	1	0.000001649	F (1, 17) = 2,527	P=0,1303
	Genotype	0.000003665	1	0.000003665	F (1, 17) = 5,616	P=0,0299
	Treatment	0.000002875	1	0.000002875	F (1, 17) = 4,405	P=0,0511
	Residual	0.00001109	17	6.526E-07		

Figure 6B

1 month

MJF14 %area Amygdala	ANOVA table	SS (Type III)	DF	MS	F (DFn, DFd)	P value
	Interaction	0,02261	1	0,023	F (1, 33) = 0,9474	P=0,3375
	Genotype	0,02864	1	0,029	F (1, 33) = 1,200	P=0,2812
	Treatment	0,4234	1	0,423	F (1, 33) = 17,74	P=0,0002
	Residual	0,7875	33	0,024		

6 months

MJF14 %area Amygdala	ANOVA table	SS (Type III)	DF	MS	F (DFn, DFd)	P value
	Interaction	0,00002504	1	0,00002504	F (1, 21) = 0,00021	P=0,9885
	Genotype	8,556E-06	1	0,000008556	F (1, 21) = 7,213e-	P=0,9933
	Treatment	1,393	1	1,393	F (1, 21) = 11,74	P=0,0025
	Residual	2,491	21	0,1186		

Figure 6C

1 month

MJF14 %area SN	ANOVA table	SS (Type III)	DF	MS	F (DFn, DFd)	P value
	Interaction	0,0003403	1	0,0003403	F (1, 21) = 0,04094	P=0,8416
	Genotype	0,0004057	1	0,0004057	F (1, 21) = 0,0488(	P=0,8273
	Treatment	0,05297	1	0,05297	F (1, 21) = 6,371	P=0,0197
	Residual	0,1746	21	0,008313		

6 months

MJF14 %area SN	ANOVA table	SS (Type III)	DF	MS	F (DFn, DFd)	P value
	Interaction	0,0006161	1	6E-04	F (1, 33) = 1,030	P=0,3176
	Genotype	0,00005385	1	5E-05	F (1, 33) = 0,09000	P=0,7661
	Treatment	0,0138	1	0,014	F (1, 33) = 23,07	P<0,0001
	Residual	0,01974	33	6E-04		

Figure 6D

1 month

p62 %area Amygdala	ANOVA table	SS (Type III)	DF	MS	F (DFn, DFd)	P value
	Interaction	0,000116	1	0,000116	F (1, 19) = 0,00162	P=0,9683
	Genotype	0,0006095	1	0,0006095	F (1, 19) = 0,0085(	P=0,9275
	Treatment	0,4415	1	0,4415	F (1, 19) = 6,164	P=0,0225
	Residual	1,361	19	0,07164		

6 months

p62 %area Amygdala	ANOVA table	SS (Type III)	DF	MS	F (DFn, DFd)	P value
	Interaction	0,00231	1	0,002	F (1, 28) = 0,3001	P=0,5882
	Genotype	0,007669	1	0,008	F (1, 28) = 0,9961	P=0,3268
	Treatment	0,2225	1	0,223	F (1, 28) = 28,90	P<0,0001
	Residual	0,2156	28	0,008		

Figure 6E

1 month

p62 %area SN	ANOVA table	SS (Type III)	DF	MS	F (DFn, DFd)	P value
	Interaction	0,00000103	1	0,00000103	F (1, 21) = 0,00456	P=0,9468
	Genotype	0,0000751	1	0,0000751	F (1, 21) = 0,3327	P=0,5702
	Treatment	0,005778	1	0,005778	F (1, 21) = 25,60	P<0,0001
	Residual	0,00474	21	0,0002257		

6 months

p62 %area SN	ANOVA table	SS (Type III)	DF	MS	F (DFn, DFd)	P value
	Interaction	0,00001757	1	2E-05	F (1, 33) = 0,03445	P=0,8539
	Genotypes	4,516E-06	1	5E-06	F (1, 33) = 0,00885:	P=0,9256
	Treatment	0,01277	1	0,013	F (1, 33) = 25,04	P<0,0001
	Residual	0,01683	33	5E-04		

Figure 6F

Pser129 %area Amygdala	ANOVA table	SS (Type III)	DF	MS	F (DFn, DFd)	P value
	Interaction	0,4314	2	0,216	F (2, 38) = 0,8626	P=0,4301
	Treatment	5,86	2	2,93	F (2, 38) = 11,72	P=0,0001
	Genotype	0,2651	1	0,265	F (1, 38) = 1,060	P=0,3097
	Residual	9,502	38	0,25		

Fig 6G

Pser129 %Area SN	ANOVA table	SS (Type III)	DF	MS	F (DFn, DFd)	P value
	Interaction	0.003383	2	0.001692	F (2, 36) = 0,3490	P=0,7078
	Treatment	0.3849	2	0.1924	F (2, 36) = 39,70	P<0,0001
	Genotype	0.005369	1	0.005369	F (1, 36) = 1,108	P=0,2996
	Residual	0.1745	36	0.004847		

Figure 7B

TH OD 1 month (%contralateral)	ANOVA table	SS (Type III)	DF	MS	F (DFn, DFd)	P value
	Interaction	152,4	2	76,19	F (2, 28) = 1,541	P=0,2317
	Treatment	1472	2	736,2	F (2, 28) = 14,90	P<0,0001
	Genotype	94,08	1	94,08	F (1, 28) = 1,903	P=0,1786
	Residual	1384	28	49,43		

Figure 7C

TH OD 6 months (%contralateral)	ANOVA table	SS (Type III)	DF	MS	F (DFn, DFd)	P value
	Interaction	57,16	2	28,58	F (2, 42) = 0,4553	P=0,6374
	Treatment	7695	2	3848	F (2, 42) = 61,29	P<0,0001
	Genotype	0,6648	1	0,6648	F (1, 42) = 0,01059	P=0,9185
	Residual	2637	42	62,78		

Figure 8B

TH %contralateral 1 month	ANOVA table	SS (Type III)	DF	MS	F (DFn, DFd)	P value
	Interaction	127,8	2	63,89	F (2, 28) = 0,8275	P=0,4475
	Genotype	59,05	1	59,05	F (1, 28) = 0,7649	P=0,3892
	Treatment	1413	2	706,6	F (2, 28) = 9,152	P=0,0009
	Residual	2162	28	77,2		

Figure 8C

TH %contralateral 6 months	ANOVA table	SS (Type III)	DF	MS	F (DFn, DFd)	P value
	Interaction	19,25	2	9,623	F (2, 43) = 0,08459	P=0,9190
	Genotype	111,5	1	111,5	F (1, 43) = 0,9799	P=0,3278
	Treatment	16624	2	8312	F (2, 43) = 73,07	P<0,0001
	Residual	4892	43	113,8		

Supplementary Figure 1A

Challening beam Total Errors/steps	ANOVA table	SS (Type III)	DF	MS	F (DFn, DFd)	P value
	Interaction	0,001788	2	0,0008942	F (2, 60) = 0,02486	P=0,9755
	Genotype	0,04319	1	0,04319	F (1, 60) = 1,201	P=0,2776
	Treatment	0,4131	2	0,2065	F (2, 60) = 5,742	P=0,0052
	Residual	2,158	60	0,03597		
**Challening beam Total steps/Sec**	**ANOVA table**	**SS (Type III)**	**DF**	**MS**	**F (DFn, DFd)**	**P value**
	Interaction	2,471	2	1,236	F (2, 61) = 0,5433	P=0,5836
	Genotype	20,16	1	20,16	F (1, 61) = 8,866	P=0,0042
	Treatment	64,69	2	32,35	F (2, 61) = 14,22	P<0,0001
	Residual	138,7	61	2,274		

Supplementary figure 1B

Challening beam Total Errors/steps	ANOVA table	SS (Type III)	DF	MS	F (DFn, DFd)	P value
	Interaction	0,3521	2	0,176	F (2, 46) = 3,123	P=0,0535
	Genotype	0,2666	1	0,267	F (1, 46) = 4,730	P=0,0348
	Treatment	0,628	2	0,314	F (2, 46) = 5,571	P=0,0068
	Residual	2,593	46	0,056		
**Challening beam Total steps/Sec**	**ANOVA table**	**SS (Type III)**	**DF**	**MS**	**F (DFn, DFd)**	**P value**
	Interaction	10,95	2	5,477	F (2, 46) = 2,710	P=0,0771
	Genotype	4,413	1	4,413	F (1, 46) = 2,184	P=0,1463
	Treatment	58,91	2	29,45	F (2, 46) = 14,58	P<0,0001
	Residual	92,95	46	2,021		

Supplementary figure 2A

Rearings per minute	ANOVA table	SS (Type III)	DF	MS	F (DFn, DFd)	P value
	Interaction	26.05	2	13.03	F (2, 45) = 1,659	P=0,2017
	Genotype	0.6933	1	0.693	F (1, 45) = 0,08829	P=0,7677
	Treatment	65.94	2	32.97	F (2, 45) = 4,199	P=0,0213
	Residual	353.3	45	7.852		

Supplementary figure 2B

Grooming (time)	ANOVA table	SS (Type III)	DF	MS	F (DFn, DFd)	P value
	Interaction	6.478	2	3.239	F (2, 45) = 0,3492	P=0,7071
	Genotype	23.88	1	23.88	F (1, 45) = 2,575	P=0,1155
	Treatment	43.29	2	21.65	F (2, 45) = 2,334	P=0,1085
	Residual	417.3	45	9.274		

Supplementary figure 2C

Forelimbs: Left and Right	ANOVA table	SS	DF	MS	F (DFn, DFd)	P value	Repeate measure
	Treatment	36083	2	18041	F (2, 46) = 10,87	P=0,0001	
	Genotype	5037	1	5037	F (1, 46) = 3,036	P=0,0881	
	Left/Right	208.5	1	208.5	F (1, 46) = 1,915	P=0,1730	
	Treatment x Genotype	4477	2	2239	F (2, 46) = 1,349	P=0,2695	
	Treatment x Left/Right	708.1	2	354.1	F (2, 46) = 3,252	P=0,0477	
	Genotype x Left/Right	170.9	1	170.9	F (1, 46) = 1,570	P=0,2165	
	Treatment x Genotype x Left/Right	214.4	2	107.2	F (2, 46) = 0,9848	P=0,3812	
	Subject	76327	46	1659			
	Residual	5008	46	108.9			

Supplementary figure 2D

Hindlimbs: Left and Right	ANOVA table	SS	DF	MS	F (DFn, DFd)	P value	Repeate measure
	Treatment	33953	2	16977	F (2, 46) = 6,622	P=0,0030	
	Genotype	4295	1	4295	F (1, 46) = 1,675	P=0,2020	
	Left/right	1802	1	1802	F (1, 46) = 17,44	P=0,0001	
	Treatment x Genotype	3621	2	1811	F (2, 46) = 0,7062	P=0,4988	
	Treatment x Left/right	365.6	2	182.8	F (2, 46) = 1,769	P=0,1819	
	Genotype x Left/right	48.83	1	48.83	F (1, 46) = 0,4725	P=0,4953	
	Treatment x Genotype x Left/right	531.7	2	265.9	F (2, 46) = 2,573	P=0,0873	
	Subject	117933	46	2564			
	Residual	4754	46	103.3			

Supplementary figure 3A

Type A (repeated measure)	ANOVA table	SS (Type III)	DF	MS	F (DFn, DFd)	P value
	Treatment	4859071	2	2429536	F (2, 27) = 5,614	P=0,0091
	Genotype	357977	1	357977	F (1, 27) = 0,8271	P=0,3712
	Hemisphere	80154	1	80154	F (1, 27) = 0,3182	P=0,5774
	Treatment x Genotype	2941611	2	1470806	F (2, 27) = 3,398	P=0,0483
	Treatment x Hemisphere	209835	2	104918	F (2, 27) = 0,4165	P=0,6635
	Genotype x Hemisphere	778317	1	778317	F (1, 27) = 3,090	P=0,0901
	Treatment x Genotype x Hemisphere	71123	2	35561	F (2, 27) = 0,1412	P=0,8690
	animal	11685605	27	432800		
	Residual	6801887	27	251922		
**Type B (repeated measure)**	**ANOVA table**	**SS (Type III)**	**DF**	**MS**	**F (DFn, DFd)**	**P value**
	Treatment	2471248	2	1235624	F (2, 27) = 1,750	P=0,1929
	Genotype	12641	1	12641	F (1, 27) = 0,01791	P=0,8945
	Hemisphere	465116	1	465116	F (1, 27) = 1,648	P=0,2101
	Treatment x Genotype	450410	2	225205	F (2, 27) = 0,3190	P=0,7296
	Treatment x Hemisphere	42136	2	21068	F (2, 27) = 0,07467	P=0,9282
	Genotype x Hemisphere	5222	1	5222	F (1, 27) = 0,01851	P=0,8928
	Treatment x Genotype x Hemisphere	91863	2	45931	F (2, 27) = 0,1628	P=0,8506
	animal	19060165	27	705932		
	Residual	7618084	27	282151		
**Type C (repeated measure)**	**ANOVA table**	**SS (Type III)**	**DF**	**MS**	**F (DFn, DFd)**	**P value**
	ANOVA table	SS	DF	MS	F (DFn, DFd)	P value
	Treatment	2101033	2	1050516	F (2, 27) = 8,157	P=0,0017
	Genotype	13191	1	13191	F (1, 27) = 0,1024	P=0,7514
	Hemisphere	177411	1	177411	F (1, 27) = 2,545	P=0,1223
	Treatment x Genotype	38081	2	19040	F (2, 27) = 0,1479	P=0,8633
	Treatment x Hemisphere	140945	2	70473	F (2, 27) = 1,011	P=0,3772
	Genotype x Hemisphere	10154	1	10154	F (1, 27) = 0,1457	P=0,7057
	Treatment x Genotype x Hemisphere	64586	2	32293	F (2, 27) = 0,4633	P=0,6341
	animal	3477077	27	128781		
	Residual	1881996	27	69704		

Supplementary figure 3B

Type A (repeated measure)	ANOVA table	SS (Type III)	DF	MS	F (DFn, DFd)	P value
	treatment	17392547	2	8696274	F (2, 46) = 17,46	P<0,0001
	Genotype	12474	1	12474	F (1, 46) = 0,02505	P=0,8749
	Hemisphere	6772484	1	6772484	F (1, 46) = 32,17	P<0,0001
	treatment x Genotype	1402247	2	701123	F (2, 46) = 1,408	P=0,2550
	treatment x Hemisphere	5128121	2	2564060	F (2, 46) = 12,18	P<0,0001
	Genotype x Hemisphere	110709	1	110709	F (1, 46) = 0,5259	P=0,4720
	treatment x Genotype x Hemisphere	99846	2	49923	F (2, 46) = 0,2371	P=0,7898
	animal	22909356	46	498029		
	Residual	9684041	46	210523		
**Type B (repeated measure)**	**ANOVA table**	**SS (Type III)**	**DF**	**MS**	**F (DFn, DFd)**	**P value**
	Row factor	2544736	2	1272368	F (2, 46) = 1,737	P=0,1875
	(AB vs CD)	1628150	1	1628150	F (1, 46) = 2,222	P=0,1429
	(AC vs BD)	9536184	1	9536184	F (1, 46) = 31,57	P<0,0001
	Row factor x (AB vs CD)	5387835	2	2693917	F (2, 46) = 3,677	P=0,0330
	Row factor x (AC vs BD)	2558867	2	1279433	F (2, 46) = 4,236	P=0,0205
	(AB vs CD) x (AC vs BD)	33488	1	33488	F (1, 46) = 0,1109	P=0,7407
	Row factor x (AB vs CD) x (AC vs BI	230764	2	115382	F (2, 46) = 0,3820	P=0,6846
	Subject	33704663	46	732710		
	Residual	13894306	46	302050		
**Type C (repeated measure)**	**ANOVA table**	**SS (Type III)**	**DF**	**MS**	**F (DFn, DFd)**	**P value**
	treatment	65183663	2	32591831	F (2, 46) = 61,37	P<0,0001
	Genotype	221,5	1	221,5	F (1, 46) = 0,0004172	P=0,9838
	Hemisphere	16459298	1	16459298	F (1, 46) = 65,30	P<0,0001
	treatment x Genotype	89282	2	44641	F (2, 46) = 0,08406	P=0,9195
	treatment x Hemisphere	8318625	2	4159313	F (2, 46) = 16,50	P<0,0001
	Genotype x Hemisphere	63736	1	63736	F (1, 46) = 0,2529	P=0,6175
	treatment x Genotype x Hemisphere	35940	2	17970	F (2, 46) = 0,07129	P=0,9313
	animal	24427658	46	531036		
	Residual	11595197	46	252070		

Supplementary figure 6B

Pser129 %area frontal cortex	ANOVA table	SS (Type III)	DF	MS	F (DFn, DFd)	P value
	Interaction	0,08823	2	0,044	F (2, 39) = 1,190	P=0,3150
	Treatment	1,274	2	0,637	F (2, 39) = 17,18	P<0,0001
	Genotype	0,04288	1	0,043	F (1, 39) = 1,157	P=0,2887
	Residual	1,446	39	0,037		

Supplementary figure 6C

Pser129 %area Thalamus	ANOVA table	SS (Type III)	DF	MS	F (DFn, DFd)	P value
	Interaction	0,002664	2	0,001	F (2, 39) = 0,2714	P=0,7638
	Treatment	0,2937	2	0,147	F (2, 39) = 29,91	P<0,0001
	Genotype	0,001963	1	0,002	F (1, 39) = 0,3998	P=0,5309
	Residual	0,1915	39	0,005		
